# Herpes Zoster in a 9-Month-Old Immunocompetent Infant

**DOI:** 10.34172/aim.35002

**Published:** 2025-11-01

**Authors:** Muath Alammar

**Affiliations:** ^1^Department of Family Medicine, Shaqra College of Medicine, Shaqra University, Shaqra, Saudi Arabia

**Keywords:** Acyclovir, Dermatomal rash, Herpes zoster, Infant, Varicella-zoster virus

## Abstract

Herpes zoster (HZ), caused by varicella-zoster virus (VZV) reactivation, is rare in infants, typically linked to maternal VZV exposure during pregnancy. This case describes HZ presentation in a healthy infant without a clear exposure history. A 9-month-old immunocompetent female presented with a two-day fever and unilateral, dermatomal rash of vesicular lesions along the C5 dermatome. Initial misdiagnoses of insect bites or eczema delayed treatment. Diagnostic tests, including Tzanck smear showing multinucleated giant cells, confirmed HZ, with normal blood counts and negative maternal VZV antibodies, suggesting subclinical primary infection. Oral acyclovir (20 mg/kg/dose, five times daily for seven days) led to full recovery without complications. This case highlights HZ as a rare but important differential diagnosis for unilateral vesicular rashes in infants. It underscores the need for research into VZV reactivation mechanisms in early childhood.

## Introduction

 The varicella-zoster virus (VZV) causes herpes zoster (HZ, or shingles) upon reactivation. The virus remains dormant in sensory neurons until reactivated by immune suppression. The condition presents as a rash with vesicular lesions on an erythematous base, with pain. Globally, HZ incidence rates range from 3 to 5 per 1,000 person-years, peaking in those over 60. In children under 10, rates are 0.74‒1.38 per 1,000 person-years. Infants under 12 months rarely develop HZ, though those infected with VZV before 2 months show higher incidence (1.0 vs 0.19 per 1,000 person-months) due to immature immunity.

 Reports suggest that HZ prevalence among Saudi adults may match global rates.^1^ Few cases are reported in Saudi children. Ashi *et al*. documented HZ in a 13-year-old from Jeddah.^2^ Alghamdi *et al*. reported HZ in a 4-year-old from Riyadh,^3^ while the youngest case is a 3-year-old from Jeddah.^4^ This case presents an atypical HZ manifestation in a healthy infant without prior varicella infection history. It emphasizes including HZ when diagnosing unilateral vesicular rashes in pediatric patients. This case adds to the limited literature on early childhood cases and highlights the need for pathogenesis research.

## Case Report

###  Patient Information

 A 9-month-old female infant with no prior medical conditions was brought to our outpatient clinic due to skin changes and mild fever developing over two days. The infant was born full-term via uncomplicated delivery, with age-appropriate developmental milestones. She had not received the varicella vaccine, typically given after 12 months. The mother reported no history of varicella during pregnancy or delivery, and no known family members or contacts had recent varicella-like illnesses.

###  Clinical History

 The mother noticed redness on the infant’s right shoulder, measuring a few centimeters. She consulted a physician, who suspected an insect bite or eczema and prescribed a topical steroid cream. After two days of cream application, the condition worsened, prompting the family to visit our clinic. The redness had developed into clusters of fluid-filled blisters (vesicles) on an inflamed base, spreading from the right shoulder to the upper arm and anterior chest wall. The mother reported that the infant was mildly feverish but had no recent injuries, insect bites, or known exposure to varicella.

###  Physical Examination

**Figure 1:**
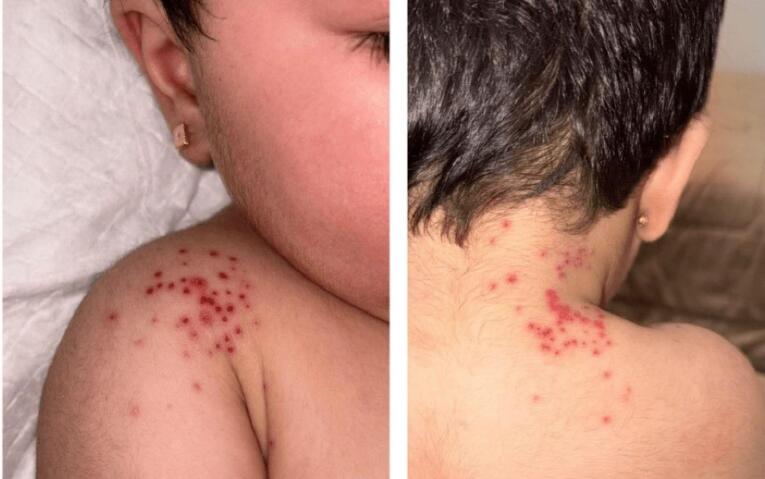
Showing Grouped Vesicles in C5 Dermatome

 The infant appeared irritable but alert and was feeding well. Her vital signs showed a temperature of 38.2 °C, heart rate of 110 beats per minute, and respiratory rate of 30 breaths per minute, all normal for her age. She weighed 10.6 kg, consistent with healthy growth for a 9-month-old. Physical examination revealed no pallor, jaundice, cyanosis, clubbing, edema, or swollen lymph nodes. Systemic assessments of cardiovascular, respiratory, abdominal, and neurological systems were normal. Dermatological examination showed tender, clear, fluid-filled vesicles grouped along the right cervical dermatome (C5), affecting the right shoulder, lateral upper arm, and anterior chest (Figure 1). No other skin or mucosal abnormalities were present, and the lesions were confined to this dermatomal pattern.

###  Diagnostic Assessment

 The patient presented with clusters of fluid-filled blisters distributed in a band-like pattern along one side of the body, suggesting a dermatomal distribution. This presentation led us to consider several causes. Initially, we considered herpes simplex virus (HSV) infection due to the vesicular lesions, but lack of mucosal involvement and clear dermatomal pattern made HSV unlikely. Impetigo was considered, but its characteristic honey-colored crusts were absent. Contact dermatitis or insect bite reactions were also considered but deemed improbable, as they do not follow dermatomal patterns. Given the patient’s history and lesion distribution, HZ (shingles) emerged as the most likely diagnosis, despite its rarity in infants. To confirm this, we conducted tests. A complete blood count revealed normal results, with a hemoglobin level of 12.4 g/dL, a total white blood cell count of 6,400 cells/mm³, and a platelet count of 340,000/mm³. A peripheral blood smear showed no abnormalities. An HIV screening test (ELISA) was negative, indicating no immune suppression. A Tzanck smear from the blister base revealed multinucleated giant cells, a hallmark of viral infections like HZ. Vesicular fluid tested positive for varicella zoster virus (VZV) DNA using real time polymerase chain reaction (PCR) targeting the ORF62 gene. No HSV-1 or HSV-2 DNA was detected. Testing the mother’s blood for VZV antibodies (IgM and IgG) showed no evidence of recent VZV infection, supporting the diagnosis in the infant.

###  Therapeutic Intervention

 The infant was diagnosed with HZ from intrauterine or early postnatal VZV exposure, despite no clear maternal infection history. Treatment began with oral acyclovir at 20 mg/kg/dose, five times daily for seven days, following pediatric guidelines. Topical fusidic acid cream was applied to prevent secondary bacterial infection. Oral paracetamol was prescribed for fever and discomfort. The mother was counselled about the condition, reassured about its benign course in healthy infants, and informed about low complication risks. She received education on medication administration and skin care for healing.

###  Follow-up and Outcomes

**Figure 2:**
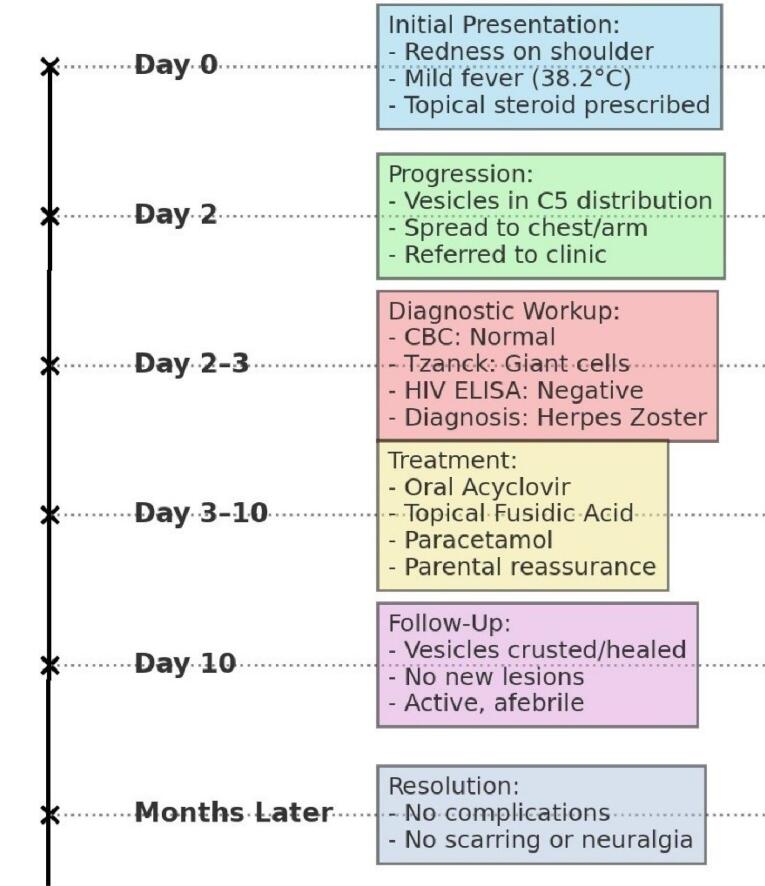
Case Timeline

**Figure 3:**
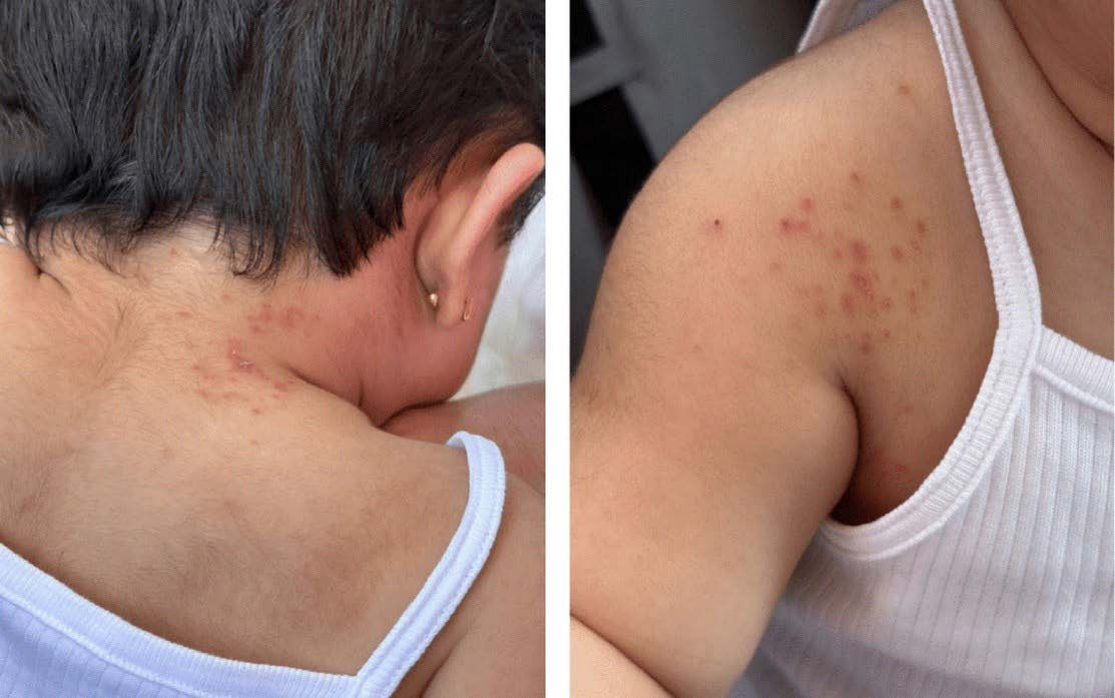
Showing the same lesion on 7th Day of Treatment

 At a follow-up visit one week later, the infant showed significant improvement (Figure 2). The vesicles had begun healing, with no new lesions appearing (Figure 3). The infant was no longer feverish, appeared comfortable, and had resumed normal activities. The treatment was well tolerated, with no adverse effects reported. She did not show any complications over the subsequent months. The mother was relieved by the clear explanation of her child’s condition and appreciated the guidance on managing symptoms, which helped alleviate her concerns about shingles in such a young child.

## Discussion

 Herpes zoster, or shingles, occurs when the VZV, dormant after chickenpox infection, reactivates. HZ is rare in infants and usually linked to maternal VZV exposure during pregnancy or infant contact with infected individuals after birth. This case of a 9-month-old immunocompetent infant with no history of chickenpox in herself or family members is unusual. The infant likely had an unrecognized VZV infection, modified by maternal antibodies that protect infants for 4–6 months after birth. As these antibodies diminish, the virus may have reactivated due to immune stress or environmental factors.

 The process of HZ in infants involves an interplay between the virus and the immune system. During chickenpox infection, VZV hides in sensory nerve cells, staying dormant through latency-associated transcripts (LATs) that help it evade immunity. Reactivation occurs when T-cell immunity weakens. In infants, the developing immune system has lower levels of CD4 + T-cells and reduced interferon-gamma production, making reactivation more likely if infection occurred *in utero* or after birth. VZV exposure *in utero* can lead to latent infection, with HZ being the first sign.^5^ Absence of a maternal chickenpox history and negative VZV antibody tests suggest a possible latent infection from an unrecognized source shedding the virus asymptomatically.

 HZ in children occurs more commonly in males and affects the thoracic or cervical dermatomes, as seen in this infant’s C5 dermatome.^6^ Pediatric HZ is rare globally, though South Asian studies report it in 0.55%–0.65% of dermatology visits, typically in adults aged 34–40 years.^7^ The youngest reported case was in a 4-day-old neonate with intrauterine VZV exposure.^8^ Pediatric HZ rates have remained stable or slightly increased post-varicella vaccination, possibly due to reduced natural immunity boosting.^9^

 Diagnosing HZ relies on its hallmark band-like rash along a single dermatome. A Tzanck smear showing multinucleated giant cells supported the diagnosis, though this test cannot distinguish between VZV and HSV. While PCR is the gold standard for confirming VZV, the Center for Disease Control and Prevention (CDC) recommends it mainly for unusual cases.^10^ Given the infant’s clear presentation, the clinical diagnosis with Tzanck smear was sufficient.

 Treating HZ in children with healthy immune systems is not always straightforward, as it often resolves independently. Starting antiviral treatment like acyclovir within 72 hours of rash onset can reduce viral activity and lower complication risks.^8^ In this case, oral acyclovir (20 mg/kg/dose, five times daily for seven days) led to full recovery with no complications like postherpetic neuralgia, which rarely affects children. More research is needed to understand VZV reactivation in infants, including the roles of silent infections, genetic factors, or environmental triggers.
